# Microbial functional genes enriched in the Xiangjiang River sediments with heavy metal contamination

**DOI:** 10.1186/s12866-016-0800-x

**Published:** 2016-08-08

**Authors:** Shiqi Jie, Mingming Li, Min Gan, Jianyu Zhu, Huaqun Yin, Xueduan Liu

**Affiliations:** 1 School of Minerals Processing and Bioengineering, Key Laboratory of Biometallurgy of Ministry of Education, Central South University, Changsha, 410083 China; 2 Department of Botany and Microbiology, Institute for Environmental Genomics, University of Oklahoma, Norman, OK 73019 USA

**Keywords:** GeoChip, Microbial functional gene, Heavy metal contamination, Metal resistance, Xiangjiang river

## Abstract

**Background:**

Xiangjiang River (Hunan, China) has been contaminated with heavy metal for several decades by surrounding factories. However, little is known about the influence of a gradient of heavy metal contamination on the diversity, structure of microbial functional gene in sediment. To deeply understand the impact of heavy metal contamination on microbial community, a comprehensive functional gene array (GeoChip 5.0) has been used to study the functional genes structure, composition, diversity and metabolic potential of microbial community from three heavy metal polluted sites of Xiangjiang River.

**Results:**

A total of 25595 functional genes involved in different biogeochemical processes have been detected in three sites, and different diversities and structures of microbial functional genes were observed. The analysis of gene overlapping, unique genes, and various diversity indices indicated a significant correlation between the level of heavy metal contamination and the functional diversity. Plentiful resistant genes related to various metal were detected, such as copper, arsenic, chromium and mercury. The results indicated a significantly higher abundance of genes involved in metal resistance including sulfate reduction genes (dsr) in studied site with most serious heavy metal contamination, such as cueo, mer, metc, merb, tehb and terc gene. With regard to the relationship between the environmental variables and microbial functional structure, S, Cu, Cd, Hg and Cr were the dominating factor shaping the microbial distribution pattern in three sites.

**Conclusions:**

This study suggests that high level of heavy metal contamination resulted in higher functional diversity and the abundance of metal resistant genes. These variation therefore significantly contribute to the resistance, resilience and stability of the microbial community subjected to the gradient of heavy metals contaminant in Xiangjiang River.

**Electronic supplementary material:**

The online version of this article (doi:10.1186/s12866-016-0800-x) contains supplementary material, which is available to authorized users.

## Background

Xiangjiang River, origined from Guangxi province, is the largest river in Hunan province, covering an area of 94,660 km^2^ and occupying 90.2 % of the total area in Hunan province [1]. Called as “the mother river of Hunan”, it takes charge of agricultural irrigation, fishery breeding, navigation, receiving pollution and supplying drinking water for residents [2]. However, numerous ores used for mining, mineral processing, and smelting of non-ferrous and rare metals are located in Xiangjiang valley [3], giving rise to serious heavy metal contamination (e.g., Cd, Cu, Zn, Pb, Hg) [4] in the river and a enormous health menace to approximately 70 million people.

Heavy metal is considered to be one of the greatest threats to the ecosystem of aquatic environment due to their high biotoxicity, perdurability and the bio-enrichment ability in food chain [5]. Heavy metals like Hg, Pb, As, Cd and Cr, which are defined as the primary toxic metals to biology, generally accumulate in soils and waters, bringing a serious diseases or even death to biosome. Plenty of studies that involving the effect of metal contamination on microbial community in aquatic ecosystem have been conducted. At present, these researches focused on the biomass and phylogenetical analysis of microbial response to heavy metal contamination. For example, the microbial communities in two sediment samples with different metal concentrations are clearly different, having bacteria in the taxa Acidobacterium (18 %), Acidomicrobineae (14 %), and Leptospirillum (10 %) in the slightly-polluted sediment and Methylobacterium (79 %) and Ralstonia (19 %) in the heavily-polluted sediment [6]. These are both well-known metal-resistant bacteria. And a previous study revealed that high pore-water Zn and As concentrations would bring the decrease of microbial biomass [7], and the enhance of sulfate reduction rates [8] suggested that metal contamination possibly has a obvious influence on the microbial abundance and activity. However, Chodak et al. [9] found that pyrosequencing demonstrated the effect of high heavy metal contents on soil microbial community measured was weak, and the abundance of most phyla stayed stable.

With regard to facing various anthropogenic and climatic fluctuations, the biodiversity of an ecosystem is pretty crucial for its functioning [10, 11]. It is widely accepted that an ecosystem whose biodiversity is higher is easier to achieve greater stability under disturbance [12, 13], because multiple communities living together can efficiently make use of available resources and work federatively to maintain the ecosystem functioning [14]. In terms of the microbial biodiversity, previous studies at contaminated sites focused on the culturable microbes [15], bacterial abundance and evenness [16, 17], sequencing of 16S rRNA genes [18–20]. There is no doubt that these approaches have been applied perfectly into the studies which concentrate on the effect of heavy metal contamination on microbial community in Xiangjiang River sediment. However, in spite of this, little is known about its functional diversity and metabolic potential at the community level, and the relationship between functional genes structure of microorganism in heavy metal polluted Xiangjiang River sediment and the environmental factors remains obscure. Therefore, in order to comprehensively know the functional gene diversity and the underlying mechanisms influencing the microbial community structure and diversity, a more integrated characterization of microbial community in this contaminated sediment is needed.

In recent years, GeoChip-based metagenomics technology, appeared as a original high throughput tool to provide significative information for the microbial community structure, composition and potential metabolic capacity, has been widely used to analyze microbial community from various habitats. For instance, GeoChip 5, containing 60,000 probes in diverse gene categories of primary microbial metabolism, such as carbon, nitrogen, sulfur, and phosphorus cycling, metal homeostasis, organic remediation, secondary metabolism, and virulence [21].

In this study, GeoChip 5.0 was employed to address two key questions. (i) What are the functional genes diversities, structures and potential metabolic capacity of microbial community in Xiangjiang River sediments with a gradient of heavy metals contaminant levels? (ii) How does the environmental variables impact the functional structure of microbial community? To answer these questions, nine sedimentary samples from three sites in Xiawan Port of Xiangjiang River (Zhuzhou city, Hunan province, China) were obtained. Our results indicated that the microbial community of the studied heavy metal contaminated sediments had a huge metabolic potential, and the functional genes structures and diversities were shaped by the heavy metal pollution.

## Results

### Geochemical description of the study sites

**Fig. 1:**
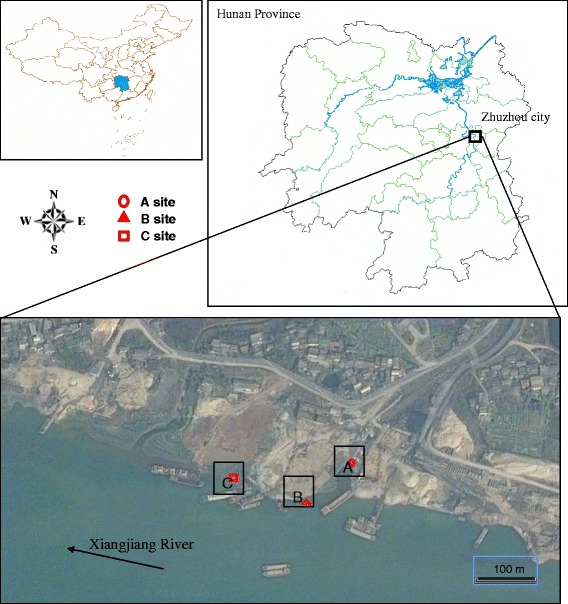
Site location and distribution of sampling points (Picture information of two parts at the top was obtained from National Geomatic Center of China. Picture information of the part at the bottom was obtained from Google, Astrium, Cnes/Spot Image and DigitalGlobe)

The different sampling locations leaded to different geochemical parameters of each site (see Fig. 1 and Table 1). There was no significant difference in pH value between three sites. While, these samples were apparently heterogeneous in terms of the content of sulfur as well as metal concentrations, such as Cu (392 ~ 570 mg/kg), Pb (383 ~ 737 mg/kg), Zn (2840 ~ 6530 mg/kg), As (177 ~ 2480 mg/kg) and Cd (23 ~ 169 mg/kg). As shown in Table 1, a majority of detected elements, including Cu, Pb, Zn, Cd, Hg, Cr and S had maximum concentration at the site A which located near the sewage outlet, and minimum content at the site C that was farthest away from the outlet. It is probable because the sewage from the factory have been diluted by the river water as the distance from drain increasing, lessening the concentration of most metal. However, As and Ni showed unlike situations when compared with above ones. It can be seen that As had the highest concentration at the site B which is 100 m away from the outlet, and the concentration of Ni at site C was higher than that of site B.

**Table 1:** Geochemical properties of sampled sediments

Site	Amount (mg/kg) in sample									pH
	Cu	Pb	Zn	As	Cd	Ni	Hg	Cr	S	
A	570	737	6530	347	169	81	24.3	88.5	5820	7.73
B	503	437	3710	2480	64	53	10.7	73.6	1420	7.78
C	392	383	2840	177	23	78	1.6	61.1	990	7.64
Reference criterion^a^	400	360	3200	14	2.1	26	0.66	72	_	_

^a^Reference with Sediment Management Standards, Chapter 173–204 WAC (2013)

All data are presented as mean value of three subsamples

### Overview of functional gene diversity

**Fig. 2:**
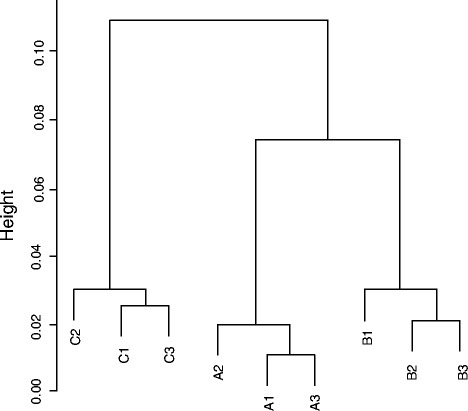
Hierarchical cluster analysis (based on Bray-Curtis distance) of functional genes in 9 studied samples from three sites named A, B, C. Every sample is named after A plant with “1”, “2” or “3” that indicates one of three replicate samples from each plant

To detailedly understand the microbial functional diversity and structure, the number of detected genes, overlapping genes between samples, unique genes, and the diversity indices were measured. A total of 25595 genes were detected, and the number of detected genes ranged from 24485 to 19431 in each of the samples. As illustrated in Table 2, the differences of the functional genes structure between samples from the same site were all less than 7 % through β-diversity calculation, showing high similarity (>90 %) among the subsamples. Nevertheless, there were apparent discrepancies between samples from different sites. The differences ranged from 13.8 % to 14.7 % between site A and site B, from 13.8 % to 14.7 % between site A and Site C, and from 18.1 to 19.4 % between site B and Sit C. Hierarchical clustering (Fig. 2) showing that the samples obtained from the same site were grouped together, and site A and site B formed a second group. The aforementioned results suggested that three sampling sites had distinct microbial functional gene structure, and adjacent sites with more similar contamination level shared the alike functional genes structure.

**Table 2:** β-diversity of studied samples

	A1	A2	A3	B1	B2	B3	C1	C2	C3
A1	0	4.6 %	3.5 %	14.2 %	13.8 %	14.3 %	21.5 %	21.8 %	21.8 %
A2		0	3.9 %	14.1 %	14.4 %	14.2 %	20.3 %	21.5 %	21.1 %
A3			0	14.7 %	13.9 %	14.1 %	20.5 %	21.6 %	21.3 %
B1				0	6.6 %	5.5 %	18.1 %	18.7 %	18.5 %
B2					0	5.4 %	18.9 %	19.4 %	19.3 %
B3						0	18.1 %	18.7 %	18.5 %
C1							0	7.0 %	5.5 %
C2								0	5.7 %
C3									0
Number of genes detected	24485	24234	24325	22206	21983	22060	19916	19431	19535

Values in table indicate the percentages of differentiations of functional gene communities among samples

As shown in Table 3, there was a high similarity (80.63 % ~ 89.64 %) of microbial community functional genes between all samples, illustrating that contamination level did not significantly affect the overall functional genes diversity. However, some differences among samples can be observed, such as samples from site A had the highest number of unique genes (2027; 8.16 %), while site C had the fewest (266; 1.31 %). In addition, Simpson’s diversity index (1/D) which was usually used for evaluating the diversity in ecology was highest in site A and lowest in site C. Similar results were observed in the Shannon index (H') with the overall diversity in the following order: A > B > C. According to the above results, it was demonstrated that the place with a higher heavy metal contamination level would have more unique genes and higher microbial functional diversity.

**Table 3:** Gene overlap, uniqueness, diversity indices, and detected gene number of studied samples

	A	B	C
A(%)	**2027(8.16 %)**	22251(89.64 %)	20015(80.63 %)
B(%)		**428(1.89 %)**	19547(85.90 %)
C(%)			**266(1.31 %)**
Number of genes detected^a^	24822	22756	20357
Simpson index (1/D)	23736.20	21436.98	19208.76
Shannon index (H')	10.0879	9.9896	9.8741
Shannon eveness (J)	0.99878	0.99870	0.99894

Values in parentheses are percentages. Boldface values indicate the number of unique genes in each sample, normal values indicate the number of overlapping genes between samples

^a^The data is the total number of detected genes in one site, including the genes which are not presented in all replicate samples

### Analysis of detected functional genes

**Fig. 3:**
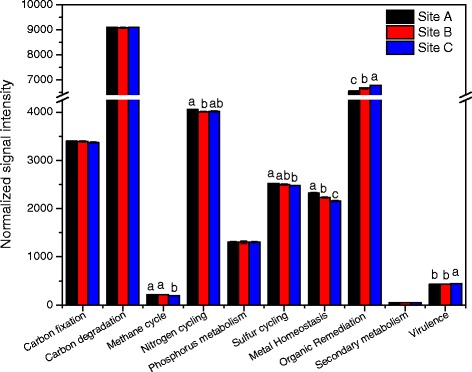
Relative richness of all functional gene group detected. The signal intensity for each functional gene category is the average of the total signal intensity from all replicates. All data are presented as mean ± SE

In these three sites, 87.28 % of the 393 functional gene included in GeoChip 5.0 was detected. The relative abundance of diverse functional gene categories were similar across all three sites (Fig. 3). Approximately 30 % of the detected probes were for genes involved in carbon degradation, another 22 % ~ 25 % were in organic remediation, about 13 % in nitrogen cycling, about 11 % in carbon fixation, 8 % ~ 10 % in sulfur cycling, 7 % ~ 8 % in metal homeostasis and few in phosphorus cycling, methane cycling, secondary metabolism and virulence. Among these, samples obtained from site A had slightly higher abundances of functional genes belonged to methane cycling, nitrogen cycling, sulfur cycling and metal homeostasis categories when compared with other sites. Further more, genes in organic remediation and virulence had the highest signal intensities at the site C.

### Detailed analysis of key functional genes

**Fig. 4:**
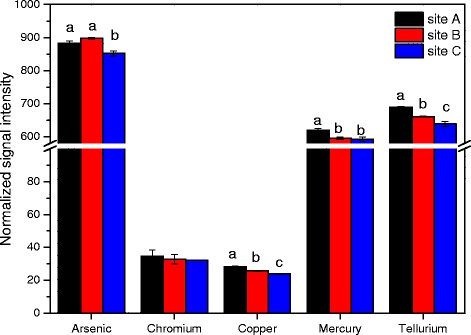
Relative abundance of detected metal resistance genes. The signal intensity for each functional gene category is the average of the total signal intensity from all replicates. All data are presented as mean ± SE

The studied sediment samples were derived from the river near the sewage outlet of a factory, leading to excessive amounts of heavy metal in sediment. In this regard, the functional genes involved in metal resistance which plays a crucial role in this ecosystem were particularly analyzed in this study. A total of 1793 ~ 1831, 1629 ~ 1595, and 1427 ~ 1379 genes involved in metal homeostasis were detected in three sites, respectively (see Additional file 1: Table S1). At the level of gene family, the normalized signal intensities of As, Hg and Te resistance genes were relatively higher among these metal (Fig. 4), for the biotoxicity of these metal are comparatively strong to microbe. In addition, it is evident that the relative abundances of Cu, Hg and Te resistance genes were highest in site A samples and lowest in site C samples. However, As and Cr resistance genes showed different conditions that there was significant difference of Cr resistance genes between three sites, and a high signal intensity of As resistance genes appeared in site B, but low intensities in site A and site C. This could be credited to the differences of metal concentrations between disparate studied sites showed in Table 1 that most of metals had high contents in site A samples, while site B had the highest concentration of As. In regard to Cr, the difference of Cr concentrations between three sites was small, and the Cr contamination level was relatively light compared with the reference criterion. A Mantel test showed that the abundance of As resistance genes was positively correlated with the As concentration (r_M_ = 0.3243, *p* = 0.018) (Table 5), and the similar condition were observed for other metals and their related genes abundances (Table 5).

**Fig. 5:**
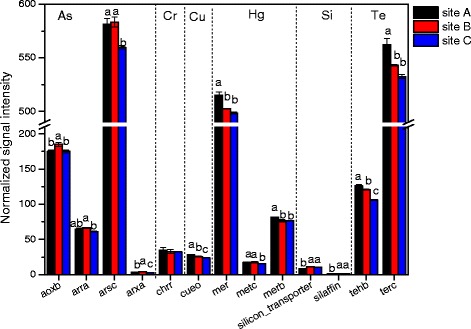
The normalized signal intensity of detected key genes involved in metal resistance. The signal intensity for each functional gene is the average of signal intensities from all the replicates. All data are presented as mean ± SE

Notably, twelve metal homeostasis genes showed differences between these three groups (Fig. 5) (*p* < 0.05), which belonged to 24 classes of *Thermoprotei, Halobacteria, Methanomicrobia, Acidobacteria, Solibacteres, Actinobacteria, Aquificae, Bacteroidetes, Cytophagia, Flavobacteria, Sphingobacteriia, Chloroflexi, Ktedonobacteria, Deinococcus, Bacilli, Clostridia, Gemmatimonadetes, Nitrospira, Planctomycetacia, Alphaproteobacteria, Betaproteobacteria, Deltaproteobacteria, Gammaproteobacteria, Eurotiomycetes*. Aoxb (arsenite oxidase), arra (arsenate respiratory reductase), arsc (arsenate reductase), arxa (aristaless related homeobox A), silicon transporter and silaffin gene were most abundant in site B. While the other six genes had the highest normalized signal intensities in site A, cueo (multicopper oxidase), mer (mercury resistance), metc(cystathionine beta-lyase), merb (organomercury lyase), tehb (tellurite resistance) and terc (tellurium detoxification). These results indicated that heavy metal contamination increased the abundance of most metal homeostasis genes, and the abundances of four genes involving As detoxification (axob, arra, arsc, arxa) had a positive correlation with the As concentration in sediment, so that the sedimentary microbiology possessing these genes can help their community adapt the heavy metal polluted environment.

**Fig. 6:**
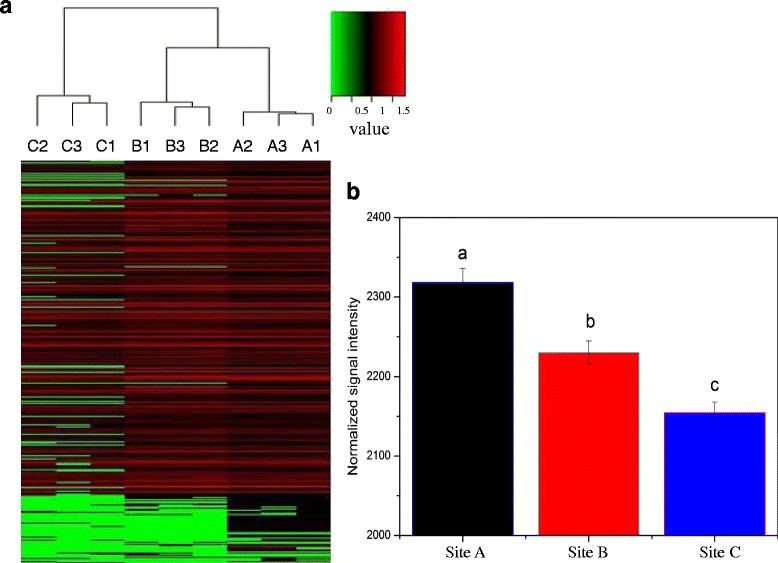
**a** Hierarchical cluster analysis (based on Bray-Curtis distance) of metal resistance genes based on hybridization signal intensities for all wells. Every sample is named after A plant with “1”, “2” or “3” that indicates one of three replicate samples from each plant. **b** Relative abundance of all detected metal resistance genes. The signal intensity for each functional gene category is the average of the total signal intensity from all replicates. All data are presented as mean ± SE

It was observed in Fig. 6a that a number of metal resistance genes were detected (1898 gene probes from all sites), which confering resistance to various metals, including Cu, Hg, Cr, As and Te. These genes were clustered by metal contamination level. In addition, the summation of the relative abundance of metal resistance genes was highest in site A (normalized signal intensity = 2321.20), then followed by site B (normalized signal intensity = 2230.11) and site C (normalized signal intensity = 2154.62) with significant difference (ANOVA, *p* < 0.05) (Fig. 6b).

**Fig. 7:**
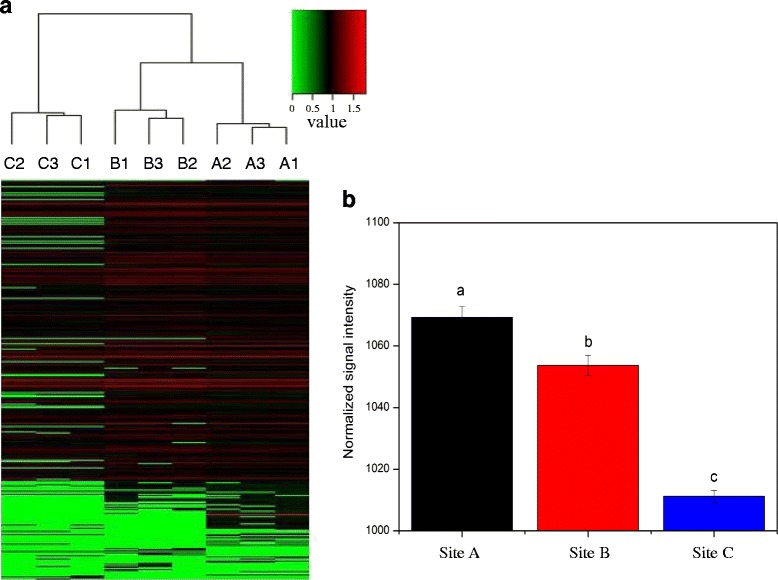
**a** Hierarchical cluster analysis (based on Bray-Curtis distance) of dsr genes based on hybridization signal intensities for all wells. Every sample is named after A plant with “1”, “2” or “3” that indicates one of three replicate samples from each plant. **b** Relative abundance of dsr genes. The signal intensity for each functional gene category is the average of the total signal intensity from all replicates. All data are presented as mean ± SE

High concentration of sulfur was also observed in these sediment samples (Table 1). Sulfur metabolism is considered to be propitious to alleviate the biotoxicity of heavy metal imposed to microbe which plays an important role in heavy metal contaminated river. Many sulphate reducing bacteria (SRB) are capable of reducing various metal [22], so those genes coding for the dissimilatory sulfite reductase (dsr) were examined. GeoChip 5.0 contains dsrA and dsrB probes to analyze the potential of sulfur reduction and sulfate-reducing bacterial populations, which were employed in this study to account for the impact of heavy metal pollution on sediment microbial functional genes structure. DsrA and dsrB genes were detected in all samples (A: 889 ~ 905; B,:795 ~ 810; C:674 ~ 694; see Table S1). Hierarchical cluster analysis of all detected dsr genes showed that subsamples were grouped together, and site A and site B formed a second group (Fig. 7a). What’s more, there was a significant difference in the relative abundance of dsr genes between three sites (ANOVA, *p* < 0.05) (Fig. 7b), and the site A with most serious heavy metal pollution had the highest signal intensity of dsr gene, which further supports the positive relationship between heavy metal contamination level and relative abundance of dsr gene. In addition, mantel test analysis showed a positive correlation between the S concentration and the abundance of dsrA and dsrB genes (r_M_ = 0.5172, *p* = 0.007), and between metal concentrations and the abundance of dsr genes (r_M_ = 0.6927, *p* = 0.013) (Table 5).

### Relationship between environment factors and functional genes

To discern the connection between environmental factors and the sediment microbial functional genes structure of Xiangjiang River sediment contaminated with heavy metal, a Mantel test was performed (Table 4). The results showed that the gradients of S, Cu, Cd, Hg and Cr were significantly correlated with the microbial community functional structure (*p* < 0.01), indicating that these metal were of pronounced importance in shaping the microbial functional genes structure of heavy metal contaminated sediment.

**Table 4:** Mantel test of the relationship of whole microbial community functional structure to individual environmental variables

Environmental variable	r_M_	P
pH	0.1835	0.124
ORP	0.1654	0.113
S	0.6039	**0.008**
Cu	0.9374	**0.001**
Pb	0.6547	0.012
Zn	0.7414	0.016
As	0.3163	0.026
Cd	0.7656	**0.005**
Ni	0.2332	0.039
Hg	0.8819	**0.001**
Cr	0.7437	**0.003**

Boldface values indicate significant *P* values (<0.01)

Further, Mantel test was performed to examine the relationships between various functional gene groups and individual metal concentration. As shown in Table 5, positive correlations between the single metal concentration and the abundance of the corresponding resistant genes were found. For instance, among these metals, chromium concentration of sediments and chromium resistant genes had a most positively relevance (r_M_ = 0.8295, *p* = 0.001), and arsenic resistant genes and arsenic concentration had the lowest correlation. All aforementioned results demonstrated that microbial community in heavy metal contaminated sediments and functional gene structures were largely shaped by the surrounding metals.

**Table 5:** Mantel test of relationship of different gene categories to corresponding environmental variables

Gene category	Environmental variable	r_M_	P
Arsenic resistant genes	Arsenic	0.3243	0.018
Chromium resistant genes	Chromium	0.8295	0.001
Copper resistant genes	Copper	0.5743	0.017
Mercury resistant genes	Mercury	0.8176	0.002
dsr genes	sulfur	0.5172	0.007
dsr genes	all studied metal	0.6927	0.013

The signal intensity of different functional genes among 9 samples was used as the first matrix; normalized related environmental variables were used as the second matrix

## Discussion

Exploring the microbial community structure, including both the microbiological compositions and functional genes structure, is of pronounced importance to deeply uncover the intricate influence of environment on microorganism. In this study, we focused on the change of sediment microbial functional genes structure caused by heavy metal contamination in river, and found that the pollution level could bring about varying degrees of impact on the functional genes diversity. The data of GeoChip 5.0 demonstrated that the site with more severe heavy metal contamination had higher diversity and more functional genes involving metal resistance, such as cueo, mer, metc, merb, tehb and terc gene. More comprehensive view of the overall functional structure and metabolic potential of sediment microbial communities was provided.

A mass of evidences suggest that microorganisms are far more sensitive to heavy metal stress than animals or plants growing on the same soils [23]. Although some kinds of metal (e.g., Fe, Cu, Zn) are essential element to microorganism which can ensure the normal growth and reproduction, high concentration of them would inhibit microbial metabolism or give rise to their death [24]. Numerous metal show a strong affinity to biological ligand, such as phosphoric acid, purine and pyrimidine, then prevent the synthesis of biomacromolecule like nucleic acid and protein. And some metal can bring damage to cytomembrane, destroying the transportation of nutrient [23]. Moreover, the impact of heavy metal on microbial community is also undisregardable, and a plenty of studies have been done to explore the mechanism of it [9, 25, 26]. The results of this study demonstrated that the microbial functional gene structure was correlated to the heavy metal contamination. As shown in Additional file 2: Figure S1, the overall functional genes of nine sediment samples from three sites were analyzed, and then samples from the same site were gathered together, while those from different sites were separated from each other. The conclusion was further supported by Fig. 3 which depicted the detailed distinctions of various gene categories among different sites, such as genes involved in methane cycling, nitrogen cycling, sulfur cycling, metal homeostasis and organic remediation.

Previous study at Xiangjiang River suggested that heavy metal contamination have ecological impact on bacterial community composition and diversity [27]. As illustrated in one research, microbial community structure was highly diverse and heterogeneous in four studied sediment samples which are obtained from Xiangjiang River (Zhu zhou) with heavy metal contamination, and α-Proteobacteria was significantly increased with the increases in heavy metal. However, the moderately polluted sediment X sample had the greatest species diversity, which is different with our observation. Another previous study illustrated that heavy metals would decrease the diversity of functional genes [28], but in this research, the site with more heavy metal had higher diversity in comparison among three studied sites (Table 3). Our results comply with a similar study about heavy metal contamination in Montana, which showed that the diversity of microbial community in sediment was evaluated across a heavy metal contamination gradient [29]. It was supposed that long term contamination has made the microbes adapted to the polluted environments, and maintained their diversity by various of resistance mechanisms [30]. To date, it is generally accepted that biodiversity plays an important role in enhancing the ecosystem stability by temporal and spatial variability [31, 32], resistance against abiotic perturbations [33], and biotic invasions [34]. Different species in diverse communities respond differentially to the environmental perturbations, making ecosystem regularly function. While, the impact of increasing metal stress on microbial diversity depends on the initial state of the system [23].

As we all know, the specific environment with high concentration of heavy metal is beneficial to screen dominant bacteria which is capable of resisting metal toxicity. The reason is believed to be correlated to the long-term natural selection which will reserve the species with ability to adapt and survive, and weed out the ones lack in the capacity. From an agricultural field subjected to Cr contamination, Maqbool et al. [35] isolated and screened twenty bacterial which can resist Cr(VI). For another example, six strains showed high degree of metal resistances were selected by Kumar et al. [36] from the soil samples collected from fly ash contaminated region near National Thermal Power Plant which had high content of various heavy metal. However, there is no inevitable relationship between the high heavy metal concentration of the studied sediment samples and the increase of metal resistant bacteria. Even though the metal content shown in Table 1 were fairly high, the amount actually effect on microorganism may be much lower, leading to little selection pressure for resistant bacteria. “Total” metal concentrations in sediment are not a good indicator of the actual concentration in the sediment to which microorganisms are exposed [23]. Only when a plenty of resistant genes corresponding to metals were detected in river sediment can we deduced that the relative resistant genes were enhanced and impacted by the high level heavy metal contamination. In this study, the relative abundance of metal resistance genes from lesser polluted site (site C) was lower than that of site A with high contamination level (Figs. 4 and 6), and the majority of metal detoxication gene involved in the array were abundant in site A (Fig. 5), confirming the influence of high concentration metal on microbial metal resistant genes.

Sulfate reducing bacteria (SRB) possess the capacity of reducing various heavy metal, such as Fe, Cu, As, Cd, Mn and others [37, 38]. SRB make use of the way of precipitation to decrease the metal availability to microorganism or alter the metal valence state and chemical speciation to lighten the metal biotoxicity, or generate metal sulfides to achieve the goal of detoxication [39, 40]. In the black amorphous sludge which was rich in sulfur, iron, aluminum, and acidity, Riefler et al. [41] found a large population of sulfur-reducing bacteria and took advantage of them to treat acid mine drainage with high concentration of diverse heavy metal. Dissimilatory sulfite reductase (dsr) is a key enzyme in SRB, which catalyses sulfite transform to sulfide with multi-steps of electronic transfer. The amount of dsr genes will influence the capacity of reducing toxic metal by SRB in river sediment [42]. In this study, higher concentration of sulfur was measured in site A (Table 1), which had the most serious heavy metal contamination, suggesting the strong ability of sulfate reduction [8]. Fig. 6 showed the relative abundance of dsr gene was positive correlated with the heavy metal contamination level, and mantel test indicated dsr genes was significantly correlated with S content (r_M_ = 0.5172, *p* = 0.007) and positively correlated with metal concentration (r_M_ = 0.6927, *p* = 0.013).

However, these results can hardly reflect the comprehensive information of metal resistant genes, since most metal homeostasis genes represented in GeoChip 5.0 are transporters which was the most common mechanism of metal resistance in bacteria, then other mechanisms including sequestration, reduction and lower the nutrient metal influx were neglected. What’s more, the suitable resistant gene probes for all heavy metal are not covered on the GeoChip, such as the resistant genes corresponding to Fe, Zn, Pb and Ni which had high concentrations in studied sediments are not included. Besides, it is notable that some metal resistant genes can work on more than one kind of metal. For instance, the *czc* operon plays crucial roles in resisting the biotoxicities of metal Zn, Cd and Co [43].

In summary, a complicated functional structure of microbial communities in Xiangjiang River sediment with heavy metal contamination was detected. Positive correlations were found between the level of metal pollution with the community functional diversity, and with the relative abundance of associated metal resistant genes. While, due to the limitation of DNA genomics, transcriptomic approaches will be a consequent step to illuminate the activity of microbial functional genes. The information of transcribed RNA will actually reflect the expression of functional genes which were directly impacted by environmental perturbation. In order to comprehensively and detailedly understand the effect of heavy metal contamination on microbial community in sediment, more systematic, in-depth analyses are needed.

## Conclusion

GeoChip 5.0 was used to analyze the microbial functional diversity and genes structure in sediments of Xiangjiang River contaminated with various heavy metal. The results showed that heavy metal contamination did not significantly impact the overall microbial functional structure, while sediment sampled from site near the sewage outlet had more unique genes and a higher microbial functional diversity in comparison with other two groups. The abundance of functional genes involved in metal resistance had a positive correlation with the level of heavy metal contamination. Notably, the relative abundance of dsr gene coding for the dissimilatory sulfite reductase had a significant difference between three sites, supporting the effect of heavy metal contamination on microbial community in sediment. S, Cu, Cd, Hg and Cr were were determined to be key factors shaping the microbial community structure. In summary, the results of markedly linkages between microbial metabolic potentials and heavy metal contamination were influential, making it possible to understand the mechanism of microorganism adapting to environmental fluctuation. Future studies were needed to to further investigate direct response of microbial community at the transcription and translation level.

## Methods

### Site describing and sample collection

Studied sediments were sampled from Xiangjiang River (N 27.8554401, E 113.0786195, Zhuzhou city, Hunan province, China), which is a tributary of the Yangtze River. For this study, a total of nine sediments (~10 cm depth) were sampled from three sites near a sewage outlet in Xiangjiang River.. At each site, three 1 m × 1 m plots were established with a distance of approximately 2 m between adjacent plots. Five to eight soil cores were collected and mixed equally to gain one sample at each plot. These three sites has different distance from the sewage outlet, leading to the distinct pollution levels. The A samples (A1, A2, A3) were collected near the sewage outlet, which would have maximum concentration of heavy metals; the B samples (B1, B2, B3) were taken from the locations which are 100m away from the sewage outlet; at the same direction, the C samples (C1, C2, C3) were obtained 200metres away from the sewage outlet. All samples were maintained on ice after collected, then stored at −80 °C for further analysis. Sediment pH was measured by a PHS-3C pH meter (Leici, China) in a 1:2.5 suspension in water [44], and the compositions of heavy metals including Cu, Pb, Zn, As, Cd, Ni, Hg, Cr in sediments was measured by ICP-AES [45].

### Microbial community DNA isolation and purification

Given that high concentration of divalent metal ion may result in premature DNA precipitation during extraction [46], the sediment samples were pre-washed with 40 mM EDTA (pH 7.2) [47]. The community DNA was extracted using the Soil DNA Kit (D5625-01; Omega Bioservices, Norcross, GA, USA) according to the manufacturer's instructions. Then DNA quality was checked by the absorbance ratios at A260/A280 and A260/A230 using a NanoDrop ND-1000 spectrophotometer (NanoDrop Technologies Inc., Wilmington, DE). Only when the A260/A280 ratio is larger than 1.7 and the A260/A230 ratio is more than 1.8, can the DNA be used for further analysis. Purified DNA was stored at −80 °C for the following DNA analysis.

### Microbial community DNA amplification, labeling, microarray hybridization, and scanning

The amplification and hybridization of community DNA were performed at Glomics Inc. (Norman, Oklahoma, USA). Approximately 100 ng of DNA was amplified employing the Templiphi kit (GE Healthcare, Piscataway, NJ, USA), with modifications of 0.1 μM spermidine and 260 ng · μl^−1^ single-stranded DNA binding protein to enhance the efficiency and reduce representational bias [48]. The amplified DNA was labeled with fluorescent dye Cy3 (GE Healthcare) by random primer, then purified with a QIAquick purification kit (Qiagen), and dried in a SpeedVac (45 °C, 45 min: ThermoSavant, Milford, MA, USA). Next, the processed DNA wass resuspended into 27.5 μl of DNase/RNase-free distilled water, and mixed with 42 μl hybridization buffer which contains 1× Acgh blocking, 1× HI-RPM hybridization buffer, 10 pM universal standard DNA, 0.05 μg/μl Cot-1 DNA, and 10 % formamide, then incubated at 95 °C for 3 min, and kept in 37 °C for 30 min.

Hybridizations process was implemented in GeoChip 5.0 arrays (60 K) at 67 °C in a Agilent hybridization oven for 24 h. Subsequently, the slides were washed by Agilent Wash Buffers at room temperature. Then the arrays using NimbleGen MS200 Microarray Scanner (Roche NimbleGen, Inc., Madison, WI, USA) at 633 nm by a laser power of 100 and 75 % a photomultiplier tube (PMT) [49]. Scanned images were quantified with the help of ImaGene® version 6.0 (BioDiscovery, Inc., Los Angeles, CA, USA). The mean signal intensity was determinted for each spot, and local background signals were automatically ducted. Then the spots that flagged as low quality by ImaGene or with a signal to noise ratio(SNR) of less than 2.0 were removed. All poor, empty, and outlier spots were removed for the further analysis [50].

### Statistical analysis

The hybridization signal was normalized by calculating the mean signal intensity across all genes on the arrays before subsequent analysis. After removing empty, poor and outlier spots, the across-array signal was normalized based on all intensities on the arrays. Then a ratio was calculated for each positive spot by dividing the signal intensity of the spot by the mean signal intensity to obtain the normalized ratio [51].

Functional genes diversity was calculated using Simpson’s reciprocal index (1/D), Shannon Weaver index (H′) and Shannon evenness(J) using R (v.2.12.0; https://www.r-project.org/). Beta diversity was figured out for comparing differentiations of functional genes communities among habitats along environmental gradient. Beta diversity estimates were calculated using presence/absence for individual genes grouped into functional categories. We chose Sorensen’s index for showing dissimilarity (Bray-Curtis dissimilarity):

$$ \upbeta =1-\left(\frac{2\mathrm{c}}{{\mathrm{S}}_1-{\mathrm{S}}_2}\right)*100\% $$

where, S_1_ = the total number of genes within a specific functional group detected in the first community, S_2_ = the total number of genes within a specific functional group detected in the second community, and c = the number of genes within a specific functional group common to both communities. The Sorensen index ranges from 0 to 1 where 1 indicates completely different communities and 0 indicates identical communities. This research use the total number of genes detected in a sample as the S number for comparing the similarity of two microbial community functional structure.

Differences in relative abundance of functional genes between various microbial communities were analyzed by a one-way analysis of variance (ANOVA) and Tukey’s test. A significance level of *p* < 0.05 was adopted for all comparisons. For whole functional genes and specific gene, hierarchical cluster was carried out with CLUSTER (http://www.eisenlab.org/) and visualized in TREEVIEW. Further more, mantel test, a statistic test of the correlation between two matrices was performed to examine the connection between functional genes communities and environmental factors.

## Additional files

Additional file 1: Table S1. Numbers of detected genes involved in metal homeostasis and dsr genes. (PDF 102 kb) Additional file 2: Figure S1. Detrended correspondence analysis (DCA) of all functional genes in three sites. (PDF 8 kb)

## References

[1] Li Z, Zhang Q, Fang Y, Yang X, Yuan Q (2010). Examining social-economic factors in spatial and temporal change of water quality in red soil hilly region of South China: a case study in Hunan Province. Int J Environ Pollut..

[2] Zhang Z, Tao F, Du J, Shi P, Yu D, Meng Y (2010). Surface water quality and its control in a river with intensive human impacts–a case study of the Xiangjiang River, China. J Environ Manage.

[3] Zhang Q, Li Z, Zeng G, Li J, Fang Y, Yuan Q (2009). Assessment of surface water quality using multivariate statistical techniques in red soil hilly region: a case study of Xiangjiang watershed, China. Environ Monit Assess.

[4] Wang L, Guo Z, Xiao X, Chen T, Liao X (2008). Song, et al. Heavy metal pollution of soils and vegetables in the midstream and downstream of the Xiangjiang River, Hunan Province. J Geogr Sci.

[5] Zhang C, Yu Z, Zeng G, Jiang M, Yang Z, Cui F (2014). Effects of sediment geochemical properties on heavy metal bioavailability. Environ Int.

[6] Kwon M, Yang J, Lee S, Lee J, Ham B, Boyanov M (2015). Geochemical characteristics and microbial community composition in toxic metal-rich sediments contaminated with Au–Ag mine tailings. J Hazard Mater.

[7] Gough H, Dahl A, Nolan M, Gaillard J, Stahl D (2008). Metal impacts on microbial biomass in the anoxic sediments of a contaminated lake. J Geophys Res.

[8] Gough H, Dahl A, Tribou E, Noble P, Gaillard J, Stahl D (2008). Elevated sulfate reduction in metal-contaminated freshwater lake sediments. J Geophys Res.

[9] Marcin C, Marcin G, Justyna M, Katarzyna K, Maria M (2013). Diversity of microorganisms from forest soils differently polluted with heavy metals. Appl Soil Ecol.

[10] Awasthi A, Singh M, Soni S, Singh R, Kalra A (2014). Biodiversity acts as insurance of productivity of bacterial communities under abiotic perturbations. ISME J.

[11] Reich P, Tilman D, Isbell F, Mueller K, Hobbie S, Flynn D (2012). Impacts of biodiversity loss escalate through time as redundancy fades. Science.

[12] Boutin C, Aya K, Carpenter D, Thomas P, Rowland O (2012). Phytotoxicity testing for herbicide regulation: shortcomings in relation to biodiversity and ecosystem services in agrarian systems. Sci Total Environ.

[13] Cardinale B, Srivastava D, Duffy J, Wright J, Downing A, Sankaran M (2006). Effects of biodiversity on the functioning of trophic groups and ecosystems. Nature.

[14] Tang B, Zhang Z, Chen X, Bin L, Huang S, Fu F (2014). Biodiversity and succession of microbial community in a multi-habitat membrane bioreactor. Bioresource Technol.

[15] Konstantinidis K, Isaacs N, Fett J, Simpson S, Long D, Marsh T (2003). Microbial diversity and resistance to copper in metal-contaminated lake sediment. Microbial Ecol.

[16] DellAnno A, Beolchini F, Rocchetti L, Luna G, Danovaro R (2012). High bacterial biodiversity increases degradation performance of hydrocarbons during bioremediation of contaminated harbor marine sediments. Environ Pollut.

[17] Zhang J, Zhang Y, Quan X, Chen S (2013). Effects of ferric iron on the anaerobic treatment and microbial biodiversity in a coupled microbial electrolysis cell (MEC)–anaerobic reactor. Water Res.

[18] Wu L, Wen C, Qin Y, Tu Q, Nostrand V, Yuan T (2015). Phasing amplicon sequencing on Illumina Miseq for robust environmental microbial community analysis. BMC Microbiol.

[19] Korehi H, Blöthe M, Schippers A (2014). Microbial diversity at the moderate acidic stage in three different sulfidic mine tailings dumps generating acid mine drainage. Res Microbiol.

[20] Zhou J, Wu L, Deng Y, Yang Y, Zhi X, Jiang Y (2011). Reproducibility and quantitation of amplicon sequencing-based detection. ISME J.

[21] Zhao M, Xue K, Wang F, Liu S, Bai S, Sun B (2014). Microbial mediation of biogeochemical cycles revealed by simulation of global changes with soil transplant and cropping. ISME J.

[22] White C, Shaman A, Gadd G (1998). An integrated microbial process for the bioremediation of soil contaminated with toxic metals. Nat Biotechnol.

[23] Giller K, Witter E, McGrath S (2009). Heavy metals and soil microbes. Soil Biol Biochem.

[24] Zhang J, Wang L, Yang J, Liu H, Dai J (2015). Health risk to residents and stimulation to inherent bacteria of various heavy metals in soil. Sci Total Environ.

[25] Wang Y, Shi J, Wang H, Lin Q, Chen X, Chen Y (2007). The influence of soil heavy metals pollution on soil microbial biomass, enzyme activity, and community composition near a copper smelter. Ecotox Environ Safe.

[26] Ancion P, Lear G, Dopheide A, Lewis G (2013). Metal concentrations in stream biofilm and sediments and their potential to explain biofilm microbial community structure. Environ Pollut.

[27] Zhu J, Zhang J, Li Q, Han T, Xie J, Hu Y, Chai L (2013). Phylogenetic analysis of bacterial community composition in sediment contaminated with multiple heavy metals from the Xiangjiang River in China. Mar Pollut Bull.

[28] Kang S, Van N, Gough H, He Z, Hazen T, Stahl D (2013). Functional gene array-based analysis of microbial communities in heavy metals-contaminated lake sediments. FEMS Microbiol Ecol.

[29] Bouskill N, Finkel J, Galloway T, Handy R, Ford T (2010). Temporal bacterial diversity associated with metal-contaminated river sediments. Ecotoxicology.

[30] Chai L, Wang Z, Wang Y, Yang Z, Wang H, Wu X (2010). Ingestion risks of metals in groundwater based on TIN model and dose–response assessment - a case study in the Xiangjiang watershed, central-south China. Sci Total Environ.

[31] Tilman D, Reich P, Knops J (2006). Biodiversity and ecosystem stability in a decade-long grassland experiment. Nature.

[32] Weigelt A, Schumacher J, Roscher C, Schmid B (2008). Does biodiversity increase spatial stability in plant community biomass?. Ecol Lett.

[33] Mulder C, Uliassi D, Doak D (2001). Physical stress and diversity-productivity relationships: the role of positive interactions. Proc Natl Acad Sci.

[34] Eisenhauer N, Schulz W, Scheu S, Jousset A (2013). Niche dimensionality links biodiversity and invasibility of microbial communities. Funct Ecol.

[35] Maqbool Z, Asghar H, Shahzad T, Hussain S, Riaz M, Ali S (2014). Isolating, screening and applying chromium reducing bacteria to promote growth and yield of okra (Hibiscus esculentus L.) in chromium contaminated soils. Ecotox Environ Safe.

[36] Kumar K, Srivastava S, Singh N, Behl H (2009). Role of metal resistant plant growth promoting bacteria in ameliorating fly ash to the growth of Brassica juncea. J Hazard Mater.

[37] Gorny J, Billon G, Lesven L, Dumoulin D, Madé B, Noiriel C (2015). Arsenic behavior in river sediments under redox gradient: a review. Sci Total Environ.

[38] Scala D, Hacherl E, Cowan R, Young L, Kosson D (2006). Characterization of Fe (III)-reducing enrichment cultures and isolation of Fe (III)-reducing bacteria from the Savannah River site, South Carolina. Res Microbiol.

[39] Jameson E, Rowe O, Hallberg K, Johnson D (2010). Sulfidogenesis and selective precipitation of metals at low pH mediated by Acidithiobacillus spp. and acidophilic sulfate-reducing bacteria. Hydrometallurgy.

[40] Kieu H, Mueller E, Horn H (2011). Heavy metal removal in anaerobic semi-continuous stirred tank reactors by a consortium of sulfate-reducing bacteria. Water Res.

[41] Riefler R, Krohn J, Stuart B, Socotch C (2008). Role of sulfur-reducing bacteria in a wetland system treating acid mine drainage. Sci Total Environ.

[42] Venceslau S, Stockdreher Y, Dahl C, Pereira I (2014). The “bacterial heterodisulfide” DsrC is a key protein in dissimilatory sulfur metabolism. BBA-Bioenergetics.

[43] Nies D (2003). Efflux-mediated heavy metal resistance in prokaryotes. FEMS Microbiol Rev.

[44] Zhang J, Wang L, Yang J, Liu H, Dai J (2015). Health risk to residents and stimulation to inherent bacteria of various heavy metals in soil. Sci Total Environ.

[45] Ramsey M, Thompson M (1987). High-accuracy analysis by inductively coupled plasma atomic emission spectrometry using the parameter-related internal standard method. Anal At Spectrom.

[46] Kejnovsky E, Kypr J (1997). DNA extraction by zinc. Nucleic Acids Res.

[47] Gough H, Stahl D (2011). Microbial community structures in anoxic freshwater lake sediment along a metal contamination gradient. ISME J.

[48] Wu L, Liu X, Schadt C, Zhou J (2006). Microarray-based analysis of subnanogram quantities of microbial community DNAs by using whole-community genome amplification. Appl Environ Microb.

[49] Cong J, Liu X, Lu H, Xu H, Li Y, Deng Y (2015). Available nitrogen is the key factor influencing soil microbial functional gene diversity in tropical rainforest. BMC Microbiol.

[50] Liang Y, He Z, Wu L, Deng Y, Li G, Zhou J (2010). Development of a common oligonucleotide reference standard for microarray data normalization and comparison across different microbial communities. Appl Environ Microb.

[51] Wu L, Kellogg L, Devol A, Tiedje J, Zhou J (2008). Microarray-Based Characterization of Microbial Community Functional Structure and Heterogeneity in Marine Sediments from the Gulf of Mexico. Appl Environ Microb.

