# Supplementary material for: Microbial functional genes enriched in the Xiangjiang River sediments with heavy metal contamination
Source: BMC Microbiol. 2016 Aug 8;16:179. doi: 10.1186/s12866-016-0800-x (PMC4976514; doi:10.1186/s12866-016-0800-x)
Supplement: Additional file 1: Table S1. — Numbers of detected genes involved in metal homeostasis and dsr genes. (PDF 102 kb) [file 12866_2016_800_MOESM1_ESM.pdf]

Table S1. Numbers of detected genes involved in metal homeostasis and dsr genes.

Gene category	Functional gene	A1	A2	A3	B1	B2	B3	C1	C2	C3
Arsenic	aoxb	150	140	148	134	130	131	118	114	112
Arsenic	arra	53	51	51	45	46	46	42	41	43
Arsenic	arsc	476	470	475	436	431	431	373	366	367
Arsenic	arsm	46	46	46	41	41	42	37	36	36
Arsenic	arxa	3	3	3	3	3	3	2	2	2
Arsenic	total	728	722	723	659	641	653	572	559	560
Chromium	chrr	25	25	25	23	23	23	21	20	20
Copper	cueo	25	23	23	19	19	20	18	17	17
Mercury	mer	412	411	411	367	362	366	329	310	324
Mercury	merg	1	1	1	1	1	1	1	1	1
Mercury	metc	13	13	13	13	11	12	10	10	10
Mercury	merb	62	62	61	53	56	57	51	50	50
Mercury	total	488	482	486	434	437	436	391	381	385
Tellurium	tehb	107	101	105	92	90	92	71	71	69
Tellurium	terc	458	452	456	402	396	399	354	349	347
Tellurium	total	565	557	561	494	486	491	425	412	416
Total		1831	1793	1818	1629	1595	1623	1427	1379	1398
Sulfur reduction	dsrA	377	373	376	344	340	343	300	294	292
Sulfur reduction	dsrB	528	516	517	466	455	458	394	385	382
Sulfur reduction	total	905	889	893	810	795	801	694	679	674
