# Supplementary figures and images for: Microbial functional genes enriched in the Xiangjiang River sediments with heavy metal contamination
Source: BMC Microbiol. 2016 Aug 8;16:179. doi: 10.1186/s12866-016-0800-x (PMC4976514; doi:10.1186/s12866-016-0800-x)

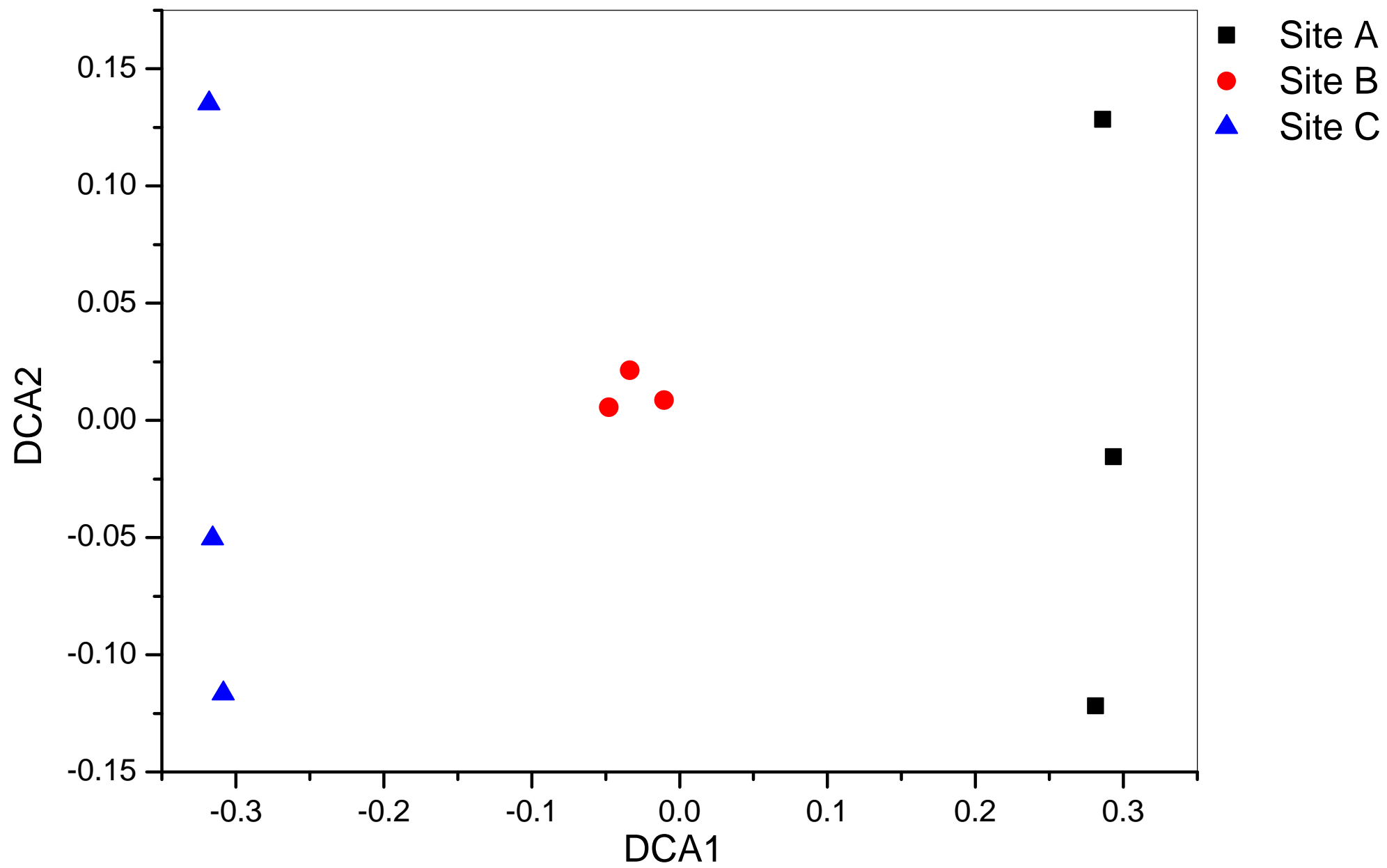

Supplement: Additional file 2: Figure S1. — Detrended correspondence analysis (DCA) of all functional genes in three sites. (PDF 8 kb) [file 12866_2016_800_MOESM2_ESM.pdf]
